# Pharmacophore-driven kinase profiling applied to the PKIS2 chemogenomic dataset

**DOI:** 10.1038/s41598-026-42945-7

**Published:** 2026-03-04

**Authors:** Ronan Bureau, Jean-Luc Lamotte, Bertrand Cuissart, Alban Lepailleur

**Affiliations:** 1 Centre d’Etudes et de Recherche sur le Médicament de Normandie, Université de Caen Normandie, Normandie Univ, CERMN UR4258, UNICAEN, CERMN, Caen, 14000 France; 2 Groupe de Recherche en Informatique, Image, Automatique et Instrumentation de Caen, Normandie Univ, ENSICAEN, CNRS, UNICAEN, GREYC UMR 6072, Caen, 14000 France

**Keywords:** Cheminformatics, Pharmacophores, Pattern mining, Isomorphism, Polypharmacology, Kinase inhibitors, Cancer, Chemistry, Computational biology and bioinformatics, Drug discovery

## Abstract

We present a data-driven and unsupervised approach for extracting 3D pharmacophore hypotheses, without prior ligand selection, from a chemogenomic kinase dataset (406 kinases and 645 compounds, PKIS2). A metric called NEM for Normalized Enrichment Measure is introduced for each pharmacophore, which quantifies change in the proportion of active compounds consistent with the pharmacophore compared to the original dataset. Based on this metric, we can identify pharmacophores associated with specific kinases and, conversely, determine all kinases that share similar metric values. This approach enables the characterization of polypharmacological profiles linked to individual pharmacophore hypotheses. We further evaluate the consistency of our results with various biological datasets, including ChEMBL, DrugBank, LINCS, KINOMEscan, and Kinobeads, showing agreement across representative case studies. This study highlights the potential of our approach for elucidating relationships between pharmacophores and kinase selectivity profiles, providing a scalable framework for exploring kinase–ligand interaction landscapes.

## Introduction

Protein kinases form a large family of enzymes that regulate cellular signaling pathways^1–3^, impacting essential functions such as cell growth, differentiation, apoptosis, and metabolism. The human kinome consists of approximately 560 kinases, classified into eight families (AGC, CAMK, CK1, STE, TK, TKL, CMGC, and atypical) based on similarities in their catalytic domains (see KinMap^4^. Information on drugs and clinical candidates targeting kinases can be accessed through resources like KLIFS^5^, the kinase structure database, which is particularly linked to PKIDB, an essential kinase database^6,7^.

In 2017, a kinase chemogenomic set encompassing 645 ATP-competitive inhibitors was published. This dataset, referred as Published Kinase Inhibitor Set 2, PKIS2^8^, succeeded an earlier version known as PKIS^9^ and covered 406 protein kinases^8^ classified into nine kinase subfamilies (AGC/Atypical/CAMK/CK1/CMGC/other/STE/TK, TKL). More recently, in 2020, a kinase chemogenomic set (KCGS) consisting of 187 highly annotated selective kinases inhibitors against 215 human kinases^10^ was published.

Pharmacophores represent the spatial arrangement of key pharmacophoric features required for ligand binding to a biological target. A recent review described the classical approaches for their definitions as well as some new trends in how pharmacophores are defined and exploited^11^. While traditionally used to characterize selective ligand–target interactions, pharmacophores can also capture common structural features shared by ligands of multiple targets. These shared pharmacophores are particularly relevant in the context of polypharmacology, where a single compound may interact with several receptors, often within the same protein family, such as kinases. Understanding these overlapping pharmacophore patterns is essential for elucidating off-target effects, designing multi-target drugs, or refining selectivity profiles in drug discovery.

Several publications discussed the concept of polypharmacology^12,13^ and pharmacophores. For example, the Polypharmacology Browser system^14,15^ performs target prediction using various molecular fingerprints, including pharmacophore fingerprints. PharmMapper^16^, a web server for drug target identification was also developed. In this case, 7302 pharmacophores models (PharmTargetDB) were derived from interaction modes between protein targets and their corresponding ligands, based on data from the Protein Data Bank^17^.

A non-exhaustive review of the literature was performed to explore existing definitions of kinase-related pharmacophores. Garuti et al.^18^ reviewed multi-kinase inhibitors, discussing their design and the potential relevance of pharmacophores, although without providing explicit pharmacophore descriptions. Reid et al.^19^ examined human tyrosine kinase inhibitors and included VEGFR pharmacophores generated by Catalyst, based on datasets of 10 and 28 compounds. A review on p38 MAP kinase inhibitors^20^ also addressed pharmacophores, focusing on their association with chemical families of inhibitors. In a study on FGFR4 inhibitors^21^, pharmacophores are described in terms of chemical functions, but not according to the classical definition of pharmacophores. Another study presented the definition of pharmacophores derived from subsets of compounds targeting eight kinases, using a QSAR-based selection approach. One publication describes the use of ECFP4 and pharmacophore fingerprints to predict affinity towards 104 kinases (ChEMBL database), using a random forest method^22^.

Given a specific target, the classical approach to defining pharmacophores involves the careful selection of a small subset of ligands known to bind the same active site with a similar binding mode. An alternative strategy defines pharmacophores based on the interaction patterns between protein targets and their ligands, as implemented in tools like PharmMapper. In our previous work, we introduced a method that automatically derives pharmacophores from a large dataset of molecules, without requiring any prior supervised selection of a ligand subset. Due to the complexity involved in covering the full conformational space of each compound, the pharmacophore elucidation problem was previously addressed using 2D topological pharmacophores^23–25^. In the present study, we further explore this issue by launching the extraction of 3D pharmacophores from multiple conformations, without any prior supervised selection of compounds. To this end, we incorporate the concept of isomorphism^26^ between molecular graphs to identify consistent spatial arrangements across conformers.

The aim of this study is to demonstrate the feasibility of mining kinase data with 3D pharmacophores extracted from PKIS2 and analyzing their relationships with affinity data (pIC_50_, pK_i_) related to kinases recorded in other datasets. These pharmacophores will be called pharmacophore hypotheses or hypotheses in the manuscript. Indeed, pharmacophores should be associated to bioactive conformations and this is not exactly the case for our study (we start from a set of conformations for each compound). Various metrics were computed for the resulting pharmacophore hypotheses, including those related to the biological profile of compounds fitting these hypotheses. Ultimately, this approach allows for the development of a pharmacophore-based kinase mining strategy, tailored to specific selective profiles.

## Materials and methods

### Chemical structure

PKIS2 included biological data of 645 compounds for 406 protein kinases. From the provided SMILES strings, a first standardization was done with the removal of salts (RDkit, Chem.GetMolFrags). Ten conformations for each compound were generated using AllChem.EMbedMultipleConfs - ETKDG version 3^27,28^. The conformations were minimized with UFFGetMoleculeForceField and classified according to the energies. The default values for the other parameters were used (EMbedMultipleConfs process). Only heteroatom hydrogens were retained, which are critical for defining the hydrophobic pharmacophoric features in our process, as detailed in our first publication^23^.

ChEMBL data for kinases were extracted from a recent version of the database^29^. Applying the filters enzyme / kinase / homo sapiens yielded 794 human kinases, 1,144,675 biological records and 461,364 IC_50_ (322,993)/ Ki(138,371) measurements. All the data associated with pChEMBL values were retained, resulting in 132,601 unique compounds with distinct Molecule ChEMBL IDs and SMILES strings. Using the same conformational generation protocol, ten conformations per compound were generated, except for 312 compounds that had fewer than 10 conformations.

### Kinase data

For PKIS2, the biological data correspond to inhibition percentages at a concentration of 1 µM. Each kinase was classified into two categories based on the biological data: active compounds (≥ 50% inhibition, class = 1) and inactive compounds (< 50% inhibition, class = 0).

### Pharmacophore hypotheses

Pharmacophore hypotheses extraction, as described in our two previous publications^23,24^, is based on a support threshold and the number of pharmacophoric features. These features correspond to generalized functionalities involved in favorable ligand-target interactions: hydrogen-bond acceptors (A) and donors (D), negatively (N) and positively (P) ionizable groups, hydrophobic areas (H), and aromatic rings (R).

In the previous version of NORNS (see Availability of data and materials for the current version), each molecule is transformed into its pharmacophore graph where vertices represent pharmacophoric features and edges encode the minimal number of bonds between the two vertices it links. In this 3D version, vertices represent pharmacophoric features and edges encode the Euclidean distances between them. Distances correspond to the minimum inter-features distance and are rounded to the nearest whole number to facilitate pharmacophoric graph comparison based on isomorphism. As an example, a distance of 5.6 Å is rounded to 6 Å. For a feature containing multiple atoms (notably an aromatic ring), the distance is defined as the minimum distance between the closest atoms associated with the two pharmacophoric features. The distance can be zero when one feature is embedded within another, such as a pyridine group with a hydrogen bond acceptor (A) inside an aromatic group (R). We retained also the textual notation for the pharmacophores^25^, listing the pharmacophoric features first followed by the distance between them.

Each compound generates pharmacophoric graphs for each conformation. With 10 conformations, the PKIS2 dataset consists of 6,450 complete pharmacophoric graphs for 645 compounds.

The order of the pharmacophore refers to the number of pharmacophoric features it contains, typically ranging from 3 to 5. Pharmacophores occurring infrequently are less relevant; thus only combinations of pharmacophoric features that meet a user-defined frequency threshold (the minimum number of compounds, i.e. support threshold) are retained. Adjusting this threshold allows control over the number of extracted pharmacophores. The PKIS2 dataset includes 86 chemotypes and setting a support threshold of 20 compounds ensures that a chemotype has at least a 25% probability of being associated with a pharmacophore.

In the pharmacophore hypothesis extraction protocol, all possible combinations of nodes that meet the support threshold are selected, and edges (distances) are subsequently incorporated for the isomorphism process. Previously, a metric called Growth Rate (GR) was assigned to each hypothesis. For a given hypothesis and a dataset partitioned into two classes, GR represents the ratio between its frequency of fit among active molecules to its frequency of fit among inactive ones. In the PKIS2 dataset, a new metric related to the Lift metric was introduced. The traditional Lift metric compares the proportion of active compounds (P_A_) in a subset (e.g., compounds associated with a pharmacophore) to the initial proportion in the overall initial dataset (P_I_). To analyze the proportions of active compounds associated with 406 kinases and track their evolution within subsets (in agreement with a hypothesis), we propose a Normalized Enrichment Measure (NEM; see Eq. 1) with a maximum value of 100, while its minimum value is negative and directly related to P_I_.

1 $$NEM=\frac{({P}_{A}-{P}_{I})}{(1-{P}_{I})}*100$$

The choice of the NEM metric was not driven by the results, but by a methodological limitation of metrics like Growth Rate (GR) or Lift. These metrics strongly depend on the baseline proportion of actives (P_I_) associated with each kinase. When P_I_ is high (e.g., kinases with many active compounds), GR and Lift artificially penalize hypotheses that are genuinely enriched because the denominator becomes large. NEM normalizes the enrichment by the maximal possible improvement above P_I_, thus allowing a fair comparison across kinases with very different P_I_ values.

**Fig. 1:**
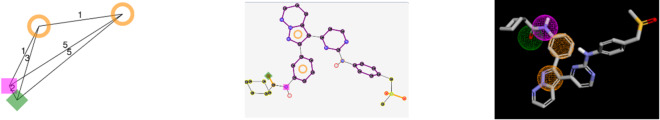
The hypothesis |D|A|R|R| |2|5|1|5|3|1| consists of two aromatic rings in dark orange, one hydrogen bond acceptor in green, and one hydrogen bond donor in magenta. The distances are displayed along with the 2D/3D representation.

Concerning the 3D representation of pharmacophore hypotheses, each pharmacophoric feature is represented as a sphere with a specific color associated with the feature: A in green, D in magenta, H in teal, R in dark orange, P in red, and N in yellow. An example is shown below, for the pharmacophore (see Fig. 1) |D|A|R|R| |2|5|1|5|3|1|, along with its 2D distance representation and the 3D representation.

Previously, we employed the MMRFS^23^ method to select a subset of hypotheses while maintaining the same statistical quality as the initial pharmacophore hypothesis set. The hyperparameters for MMRFS were set as follows: a coverage threshold of 2, a minimum of 5 new positive instances, and a maximum confidence level of 0.99. In this MMRFS study, hypotheses were classified and selected based on three criteria: NEM values, support values, and non-redundancy. All pharmacophores are first ranked by decreasing NEM and support. The top-ranked pharmacophore is selected as the first representative model. Additional pharmacophores are selected iteratively if they (i) are the highest-ranked unselected model and (ii) contribute at least five molecules that have not been included more than twice in the previously selected models. This constraint prevents redundancy between closely related patterns and ensures that each selected pharmacophore brings new chemical information. The selection is entirely automatic and reproducible.

**Fig. 2:**
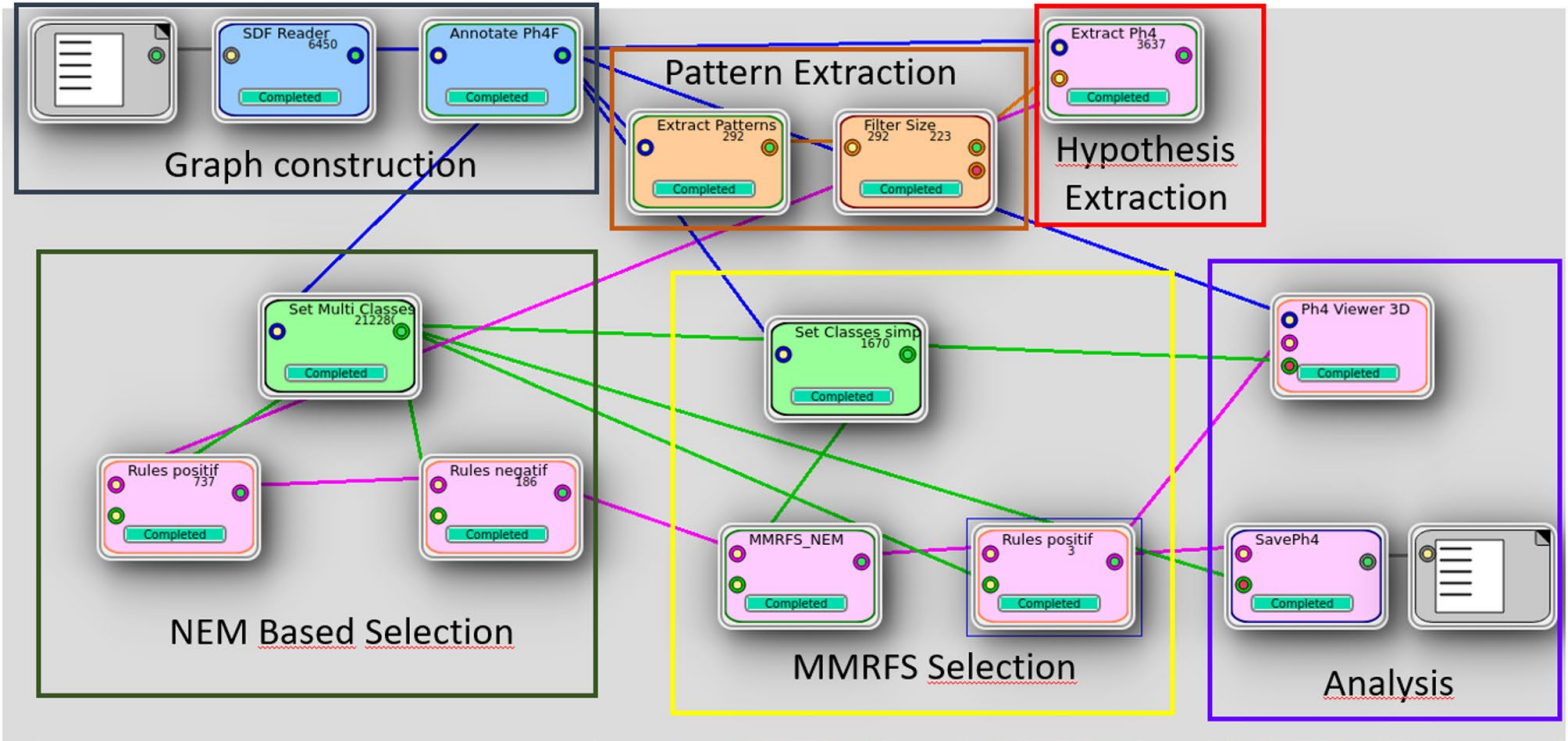
Workflow of the hypothesis extraction and selection process.

The pipeline (see Fig. 2) consists of six main stages: (i) graph construction from annotated SDF files, (ii) pattern extraction from multi-conformer graphs, (iii) hypothesis extraction, (iv) NEM-based filtering, and (v) MMRFS-based selection of representative hypotheses followed by (vi) analysis and visualization. Each module corresponds to a computational step implemented in the software, and arrows indicate data flow between modules.

## Results

### Kinase data (PKIS2)

The average number of active compounds per kinase was 52 with a standard deviation of 36. Four kinases have more than 200 active compounds: PDGFRB (CAMK), MEK5 (STE), KIT (TK), YSK4 (CMGC). Conversely, nine kinases have fewer than 10 active compounds (MAPKAPK2 (CAMK), PLK2 (Other), NEK4 (Other), MKK7 (STE), ASK1 (STE), MYO3B (STE), MAPKAPK5 (CAMK), YANK1 (AGC), ASK2 (STE)). A correlation matrix between kinases was generated using the Jaccard Score from sklearn.metrics, revealing 42 kinases highly correlated with ABL2, 39 with FRK et LCK, 37 with LYN and 34 with RET. A total of 86 kinases showed no correlation (see SI for more information).

### Pharmacophore hypotheses

The dataset consists of 6,450 3D complete graphs, corresponding to 645 compounds (each with 10 conformations). Table 1 presents the number of hypotheses extracted from this dataset and the number of compounds covered as a function of their order (i.e., the number of pharmacophoric features for each hypothesis). The order range between 4 and 5 was identified as the most relevant, considering both the number of hypotheses which is three times lower than for orders between 3 and 5, and the high coverage of 99.2% of molecules. Our goal is to identify frequent pharmacophoric determinants across thousands of kinase inhibitors. These patterns correspond to the smallest topological units that can be consistently shared across diverse chemotypes. Increasing the number of features (to 6 and 7 for instance) reduces the number of molecules matching each hypothesis and greatly expands the combinatorial search space, producing extremely sparse patterns that are not so informative for global polypharmacology analysis.

**Table 1:** Hypotheses and molecules covered as a function of the orders.

Orders	Number of hypotheses	Compounds covered in %
3–5	9978	100%
4–5	3637	99.2%
5	938	86%

We focus on hypotheses associated with a higher proportion of active compounds compared to the initial proportion in the dataset. As part of our analysis, we compared two key metrics, Lift and NEM, across different kinases scenarios: one where hypotheses are associated with 80% active compounds for a given kinase and another where the proportion is 50% (see Table 1). In the case of hypotheses with 80% active compounds, Lift values exhibit significantly greater variation compared to NEM, which remains more stable taking the P_I_ value into account. Conversely, when hypotheses contain 50% active compounds, NEM values show increased variation, with a more pronounced impact on kinases that initially have a high P_I_ value (~ 200 actives). Despite these variations, we have chosen to use NEM as the primary metric for selecting pharmacophores (Table 2).

**Table 2:** Evolution of the LIFT and NEM values as a function of P_A_ (proportion of active compounds) and P_I_ (initial proportion of active compounds).

*P*_A_/*P*_I_	LIFT/NEM	*P*_A_/*P*_I_	LIFT/NEM
0.8/0.015	53.5/80	0.5/0.015	33.3/49
0.8/0.086	9.3/78	0.5/0.086	5.81/45
0.8/0.31	2.58/71	0.5/0.31	1.61/28

For a support threshold of *n* = 20, differences between P_A_ and P_I_ were evaluated using a two-proportion z-test^30^. For the minimal support threshold (*n* = 20) and P_A_ = 0.8, statistical significance was extremely strong for all representative P_I_ values: P_I_ = 0.015 (*p* < 10^-180^), P_I_ = 0.086 (*p* < 10^-29^), P_I_ = 0.31 (*p* = 2.1 × 10^-6^). For P_A_ = 0.5, statistical significance depended on P_I_: P_I_ = 0.015 (*p* < 10^-71^) and P_I_ = 0.086 (*p* = 2 × 10^-12^) showed extremely strong enrichment, while P_I_ = 0.31 yielded no significant difference (*p* = 0.065) for *n* = 20. Our selection criteria will be based on a NEM value of 50 (*vide infra*). For P_I_ = 0.31, a NEM value of 50 corresponds to P_A_ = 0.655. With a minimal support of *n* = 20, this enrichment was statistically significant even for P_I_ = 0.31 (*p* = 8.5 × 10^-4^).

With our approach, given the relationship between pharmacophore hypotheses and kinases, we consider that whenever a compound fits a given hypothesis, it has the potential to exhibit affinity for the kinases associated with the hypothesis. PKIS2 provides data on 406 kinases, meaning that for each pharmacophore, 406 NEM values were computed. Table 3 presents the number of hypotheses, the number of kinases, and the top ten kinases ranked by the number of associated hypotheses, based on their NEM values. By focusing on the highest NEM values, we observed that out of the 3637 hypotheses, 602 exhibited at least one NEM value ≥ 80 for a given kinase, covering a total of 59 kinases.

To assess the potential of our approach, we made specific analytical choices regarding the NEM value threshold and selected a single kinase for initial focus. Using thresholds of NEM ≥ 80 or 50, DDR1 kinase^31^ ranks first in terms of the number of associated hypotheses (see Table 3). DDR1 has an initial P_I_ value of 0.26, based on 167 active compounds. DDR1 has been identified as a therapeutic target in one of the most challenging malignancies: pancreatic cancer^32^.

**Table 3:** Number of pharmacophores (P) and Kinases (K)/Top 10 kinases (number of pharmacophores ) as a function of the NEM values.

NEM ≥ 80	NEM ≥ 50	NEM ≥ 20	NEM ≥ 0
602 P/59 K	2148 P/178 K	3599 P/381 K	3637 P/406 K
DDR1 (191)	DDR1 (737)	AURKC (2012)	AURKC (3044)
MEK5 (133)	MEK5 (677)	MEK5 (1975)	WNK2 (2831)
PDGFRB (118)	PDGFRB (671)	HPK1 (1687)	AURKA (2801)
KIT (113)	KIT (557)	EPHB6 (1659)	MEK5 (2788)
PDPK1 (64)	STK36 (430)	PIP5K2C (1594)	EPHB6 (2738)
MYLK2 (54)	EGFR (399)	PDGFRB (1556)	HPK1 (2734)
LOK (48)	LOK (378)	KIT (1425)	PIP5K2C (2681)
p38-alpha (48)	CSF1R (369)	TRKA (1317)	ADCK4 2665)
EGFR (45)	PIP5K2C (365)	ADCK4 (1237	LIMK1 (2660)
PDGFRA (43)	DDR2 (355)	DDR1 (1232)	ULK3 (2600)

### DDR1 pharmacophores (PKIS2)

Starting from the 737 hypotheses with NEM ≥ 50 for DDR1 (see Table 3), we identified a set of 11 MMRFS 3D hypotheses (see Table 4, hypotheses **1**–**11**). These hypotheses cover 61% of active compounds and 13% of inactive compounds for DDR1 (P_I_ = 26%). A P_A_ values around 61% has strong statistical significance (*p* < 10⁻¹², two-proportion z-test). These hypotheses represent minimal yet recurrent 3D arrangements shared across diverse DDR1-active chemotypes. The Table 5 describes for each hypothesis all the kinases for which NEM values is ≥ 50.

**Table 4:**
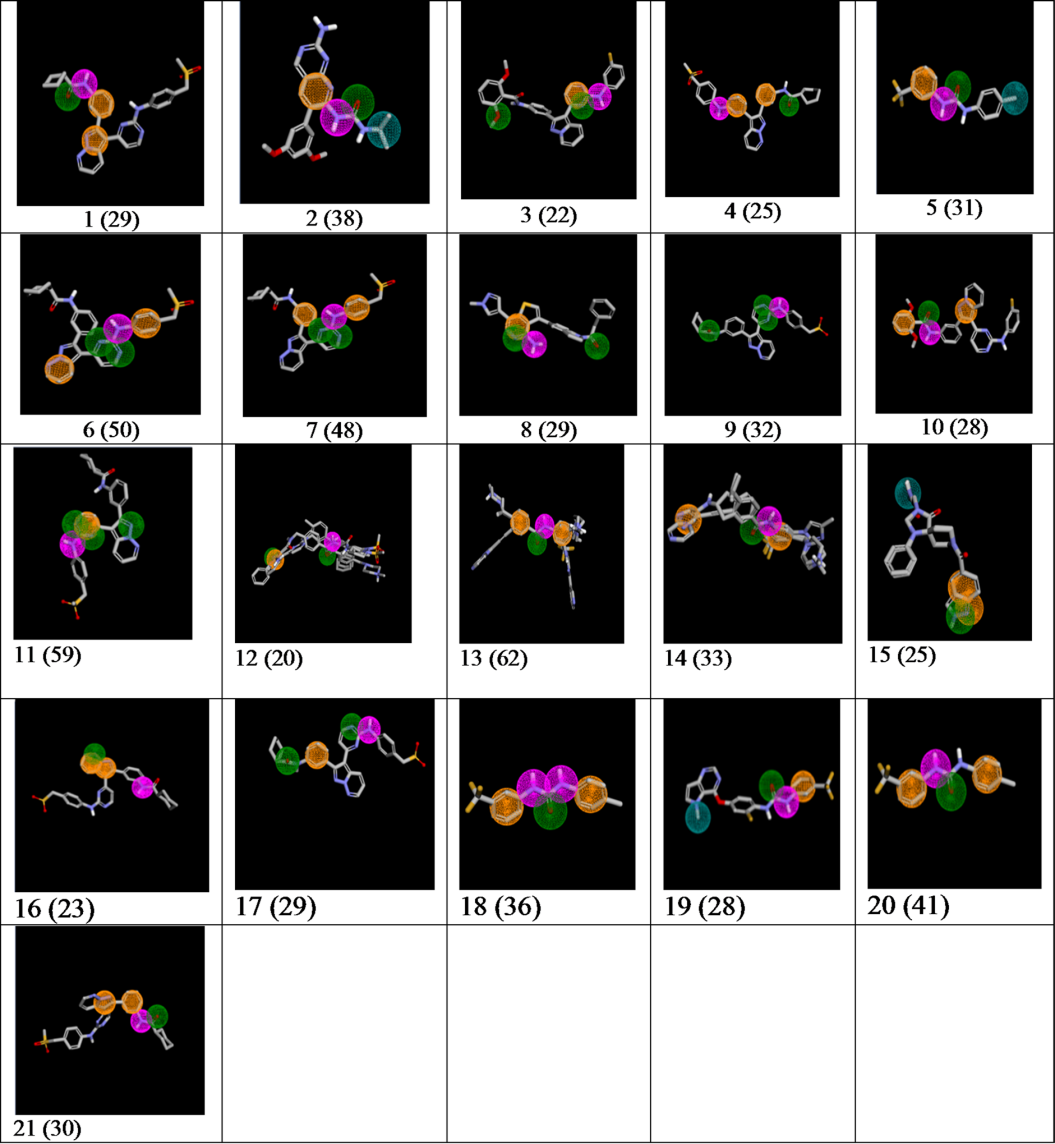
Pharmacophore hypotheses, along with their support.

**Table 5:** Kinases matching the hypotheses with NEM values ≥ 50 (NEM values between bracket).

1	CSF1R(61), DDR1(86), DDR2(65), KIT(74), PDGFRB(83), RAF1(68)
2	ABL1-nonP(50), AXL(53), BRAF(55), CSF1R(80), DDR1(86), DDR2(77), FLT1(68), FLT3(62), FLT4(60), FRK(61), KIT(84), LCK(64), LOK(70), MERTK(54), PDGFRA(69), PDGFRB(78), RAF1(73), RET(71), TIE1(53), VEGFR2(54)
3	CSF1R(54), DDR1(69), EPHB6(59), FRK(53), KIT(66), MEK5(50), PDGFRB(63), RET(55)
4	AURKC(70), BLK(51), BRK(51), CSF1R(54), DDR1(68), DDR2(55), ERBB4(50), FGR(53), FLT1(76), FLT3(68), FLT4(53), FRK(59), FYN(50), KIT(82), LCK(54), LOK(80), MEK5(81), MERTK(63), PDGFRB(93), RET(70), RIPK2(51), RSK1(Kin.Dom.1-N-terminal)(57), SLK(61), STK36(52), TNK1(68), YES(51)
5	CSF1R(51), DDR1(65), KIT(76), PDGFRB(57), RAF1(55)
6	DDR1(65), DDR2(52), EGFR(50), FGR(58), FLT3(55), FYN(55), KIT(61), LCK(57), MEK5(68), PDGFRB(67), PIP5K2C(55), RIPK2(54), STK36(52)
7	DDR1(64), FLT3(56), KIT(56), LCK(55), MEK5(64), PDGFRB(66), PIP5K2C(50)
8	AURKB(57), AURKC(56), AXL(50), CSF1R, (65) DDR1(58), EPHB6(69), KIT(84), LOK(52), MEK5(62), PDGFRA(55), PDGFRB(83), TIE1(63)
9	CSF1R(56), DDR1(58), DDR2(53), FLT1(51), LOK(57), MEK5(51)
10	DDR1(57), PDGFRB(59)
11	DDR1(52)
12	AURKB(50), CDK11(51), CSF1R(62), DDR1(66), DDR2(68), FLT1(63), FLT3(54), FLT4(59), KIT(85), LOK(62), PDGFRA(74), PDGFRB(59), RET(63), TIE1(53), VEGFR2(71)
13	CSF1R(51), DDR1(52), KIT(76), PDGFRB(74), RAF1(50)
14	CDK8(54), CSF1R(57), DDR1(63), DDR2(62), FLT1(67), FLT3(52), FLT4(54), KIT(64), LOK(54), PDGFRA(65), PDGFRB(60), RET(55), TIE1(53), TNNI3K(50), VEGFR2(65)
15	DDR1(68), KIT(52)
16	ABL1-nonP(59), ABL2(51), BRAF(75), CSF1R(78), DDR1(82), DDR2(67), FLT1(73), FLT4(69), FRK(65), FYN(51), HCK(50), KIT(87), LCK(67), LOK(67), LYN(50), MEK5(59), PDGFRA(83), PDGFRB(92), RAF1(88), RET(73), VEGFR2(60)
17	AURKC(69), AXL(59), BLK(54), BRAF(52), CSF1R(82), DDR1(81), DDR2(74), EPHB6(51), FLT1(83), FLT3(68), FLT4(60), FRK(57), KIT(84), LCK(61), LOK(87), MEK5(78), MERTK(64), PDGFRA(64), PDGFRB(89), RAF1(64), RET(74), SLK(53), STK36(51), TIE1(59), VEGFR2(56), YES(50), ZAK(60)
18	CDK8(51), CIT(54), CSF1R(79), DDR1(92), DDR2(82), FLT1(59), FLT3(60), FLT4(51), KIT(100), LOK(58), PDGFRA(64), PDGFRB(90), RAF1(62), RET(52), TIE1(50), TNNI3K(54), VEGFR2(55)
19	ABL1-nonP(52), CSF1R(73), DDR1(71), DDR2(69), FLT1(65), FLT3(58), FLT4(58), FRK(51), KIT(73), LCK(55), LOK(68), MERTK(50), PDGFRA(58), PDGFRB(65), RET(65), TIE1(62), VEGFR2(55)
20	CIT(53), CSF1R(72), DDR1(83), DDR2(75), FLT1(55), FLT3(58), KIT(89), LOK(54), PDGFRA(59), PDGFRB(80), RAF1(58), TIE1(51), TNNI3K(52), VEGFR2(52)
21	CSF1R(62), DDR1(82), DDR2(62), KIT(75), PDGFRB(84), RAF1(66)

Using the same NEM threshold, these eleven hypotheses were also found to be associated with 37 kinases (see Table 6). Among these 37 kinases, 68% belong to the TK family, whereas TK represent only 21% of the PKIS2 panel (87/406). This corresponds to a strong and statistically significant enrichment for TK (hypergeometric test followed by Benjamini–Hochberg FDR correction^33^: expected 8 TK, observed 26 TK, *p* = 1.9 × 10^-11^, FDR-corrected q = 1.1 × 10^-10^). This finding provides a pharmacophore hypothesis-based view of the selectivity profile of DDR1 ligands. To support this profile, we analyzed external datasets that show good agreement with the observed selectivity.

**Table 6:** From top left to bottom right: kinase gene symbols with family/number of active/number of inactive (in parentheses).

KIT (TK/106/30)	LCK (TK/53/30)	FLT4 (TK/35/18)	EGFR (TK/29/21)	BLK (TK/15/10)
**PDGFRB** (TK/106/36)	**PDGFRA** (TK/47/19)	PIP5K2C (Atypical/35/19)	STK36 (Other/28/27)	RSK1(Kin.Dom.1-N-terminal) (STE/15/10)
**DDR1** (TK/103/63)	**RET** (TK/46/20)	AURKC (Other/33/16)	**ABL1-nonphosphorylated** (TK/24/14)	BRK (TK/14/11)
**CSF1R** (TK/73/46)	**RAF1** (TKL/43/26)	**EPHB6** (TK/33/13)	**BRAF** (TKL/23/15)	**ERBB4** (TK/14/11)
**DDR2** (TK/ 67/40)	**FLT1** (TK/42/23)	**FGR** (TK/32/23)	VEGFR2 (TK/23/15)	**YES** (TK/14/11)
MEK5 (STE/64/33)	**TIE1** (TK/42/24)	MERTK (TK/32/21)	AURKB (Other/19/10)	
**FLT3** (TK/58/25)	AXL (TK/39/27)	RIPK2 (TKL/31/24)	**TNK1** (TK/18/7)	
**LOK** (STE/57/31)	**FRK** (TK/38/28)	**FYN** (TK/30/25)	**SLK** (STE/17/8)	

Kinases with a recorded positive biological affinity for DDR1 ligands in KINOMEscan are shown in bold.

Regarding DDR1, the compound with the highest affinity based on IC_50_ values is Dasatinib (CHEMBL1421), with an IC_50_ of 0.5 nM for DDR1. It also exhibits affinities for KIT (5 nM), PDGFRB (28 nM), CSF1R (5 nM), and DDR2 (2 nM). Additionally, it shows high affinities for LCK (0.4 nM), FYN (0.2 nM), ABL1 (1 nM), and YES (0.5 nM), all of which belong to the TK family. Dasatinib matches hypothesis **5** (*CSF1R*,* DDR1*,* KIT*,* PDGFRB*, RAF1) and hypothesis **8** (AURKB, AURKC, *AXL*, *CSF1R*,* DDR1*, EPHB6, KIT, LOK, MEK5, *PDGFRA*, *PDGFRB*, TIE1) with italicized kinases indicating those for which activity has been recorded. A recent article in *Science*^34^ describes Dasatinib as having affinities for 66 kinases based on Kinobeads data^35^. The apparent dissociation constant (K_d_^app^) confirms its strong interaction with ABL1, DDR1, DDR2, KIT, PDGFRB, FYN, YES, and LCK, among others in the previous list, as well as with MEK5 (K_d_^app^ = 1432).

We also explored the selectivity profile of the five compounds identified as DDR1 ligands (ALW-II-38-3, ALW-II-49-7, QL-XI-92, WZ-4-145, WZ-7043) in HMS LINCS database (NIH LINCS program^36,37^. Based on KINOMEscan data for these five compounds^38^, kinases for which the “percent of control” is ≤ 30% were considered active for a given compound. Among the 37 kinases identified in PKIS2, 26 (70%) are also present in the kinase list for these five compounds (see Table 6).

With these eleven hypotheses, a virtual screening was performed on the 132,601 compounds associated with kinase data in ChEMBL. A total of 35,291 compounds were found to fit at least one of the eleven hypotheses, representing 26% of the overall dataset and covering 437 kinases with at least one associated compound. This represents a significant proportion, indicating that these hypotheses likely capture key structural features associated with kinase ligands. Regarding the top five kinases matching at least one of the MMRFS-derived DDR1 hypothesis and their corresponding results in ChEMBL, only KIT exhibited a similar percentage of compounds fitting these pharmacophores, closely aligning with PKIS2 (see Table 7).

**Table 7:** Hit percentages for PKIS2 and ChEMBL (top five kinases in PKIS2).

	KIT	PDGFRB	DDR1	CSF1R	DDR2
PKIS2	49%	42%	61%	53%	51%
ChEMBL	41%	25%	24%	26%	25%

Table 8 presents the top ten kinases identified through the virtual screening on ChEMBL ranked according to the number and percentage of compounds matching the selected hypotheses. Vascular endothelial growth factor receptor 2 (VEGFR2) ranked first in terms of the absolute number of matching compounds, with 3,069 hits. Insulin-like growth factor I receptor (IGF1R) ranked first for the percentage of compounds. Based on these results, we decided to conduct the same analysis for VEGFR2 as was done for DDR1. Another motivation for this choice was the opportunity to explore a new screening strategy by applying Boolean logic to NEM-based criteria across multiple kinases for pharmacophore selection. In this case, the goal was to isolate DDR1-related hypotheses that do not align with the VEGFR2 profile.

**Table 8:** Top 10 kinases matching DDR1 pharmacophore hypotheses, ranked by the percentage of compounds fitting these pharmacophores.

Name	Family	Count	Min	Max	Percent
Insulin-like growth factor I receptor	TYR	1589	7.56	10.3	63
Serine/threonine-protein kinase B-raf	TKL	1921	7.60	10.15	47
MAP kinase ERK2	CMGC	1445	8.00	11	45
Nerve growth factor receptor Trk-A	TYR	1635	7.18	9.66	45
Tyrosine-protein kinase BTK	TYR	1527	7.82	10.85	44
Tyrosine-protein kinase SYK	TYR	1257	7.62	10.52	36
Serine/threonine-protein kinase Aurora-A	Other	1165	7.39	10.92	36
Vascular endothelial growth factor receptor 2	TYR	3069	7.04	10.7	33
Epidermal growth factor receptor EGFR	TYR	2701	7.36	11	33
Tyrosine-protein kinase JAK2	TYR	1216	7.36	10.71	18

Count: Number of compounds; Family: kinase family; Min: Minimum activity; Max: Maximum activity ; Percent: percentage of compounds fitting the pharmacophore.

Regarding IGF1R, we investigated external data to explore a potential dual biological response involving both DDR1 and IGF1R. ChEMBL1957 (IGF1R) data show 20 compounds listed as approved drugs or clinical candidates. In function of the data, six small molecules were analyzed (antibodies were notably discarded). Only two were found to fit at least one of the hypotheses, including XL-228. XL-228 is currently in Phase 1 and matches three out of the eleven pharmacophores. Based on Kinobeads data^35^, XL-228 has K_d_^app^ for 73 kinases^34^ with 753.68 for IGF1R and 220.73 for DDR1.

From hypotheses **1–11**, we performed a screening on the ligands from the KLIFS^5^ database (4,731 ligand/kinase PDB complexes extracted). As described on their website, KLIFS^39^ is a kinase database that analyzes experimental structures of catalytic kinase domains and how kinase inhibitors interact with them. Among the 4,731 ligand/kinase complexes in KLIFS, 17 are associated with DDR1_human. However, none of the ligands in these complexes matches the hypotheses. Therefore, we tested the initial set of 737 DDR1 pharmacophores (prior to MMRFS selection) to evaluate whether some of them could match the 17 DDR1_human complexes. Out of the 737 hypothesis, 464 fit at least one of the 17 ligands, and all ligands can be covered by at least 12 pharmacophores. Four pharmacophores (12–15) that cover 70% of the ligands for DDR1 complexes are shown in Table 4.

**Fig. 3:**
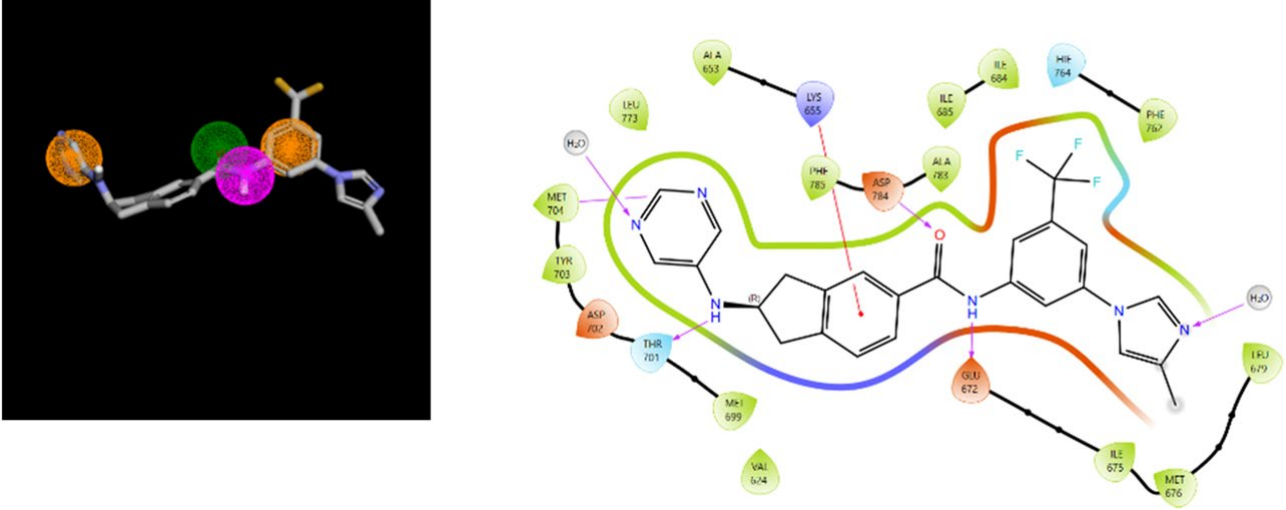
Hypothesis **14** fitted with ligand GKB (left). Ligand-protein interactions for the 6HP9 complex generated using the Protein Preparation Workflow and the ligand interaction diagram in Maestro (Schrodinger Inc)).

One DDR1 ligand, 2-amino-2,3-dihydro-1 H-indene-5-carboxamide^32^, fits the hypothesis **14** and corresponds to the PDB complex 6HP9 (GKB code for the ligand). The fit between the ligand and the hypothesis is illustrated below with a 2D depiction of the complex (Fig. 3). Two hydrogen bonds involving residues D784 and E672 are particularly observed, in agreement with the hypothesis and consistent with a type II kinase inhibition mode^40^(interactions with the Asp of DFG-out conformation and with the Glu of the αC-helix).

### VEGFR2 pharmacophore hypotheses (PKIS2)

Among the eleven hypotheses associated with DDR1, hypothese **2** is also associated with VEGFR2. In the ChEMBL dataset, VEGFR2 ranked first in terms of the number of compounds matching these eleven hypotheses. This can be partly attributed to the large volume of VEGFR2-related data in ChEMBL. Given these observations, we decided to apply the same analytical approach to VEGFR2 as the one previously carried out for DDR1.

109 hypotheses with NEM values ≥ 50 for VEGFR2 were initially identified leading to five MMRFS VEGFR2 hypotheses **16–20** (see Tables 4 and 5).

Hypotheses **16–20** covered 50% of active compounds and 6.4% of inactive compounds for VEGFR2. This represents a significant increase compared to the eleven DDR1 hypotheses previously identified for PKIS2 (26%). These five hypotheses are associated with 35 kinases (see Table 9), representing 19.6% of the 178 kinases with at least one pharmacophore having NEM ≥ 50 (see Table 9). Among these 35 kinases, 65% belong to the TK family (versus 21% in PKIS2). This enrichment is highly significant (hypergeometric test followed by Benjamini–Hochberg FDR correction^33^: expected 7.5 TK, observed 24 TK, *p* = 3.2 × 10^-10^, FDR-corrected q = 1.9 × 10^-9^). In terms of selectivity, the top five kinases remain the same as those identified with the DDR1 hypotheses **1–11** (see Table 9).

**Table 9:** From top left to bottom right: kinase gene symbols with family/number of active/number of inactive (in parentheses).

KIT (TK/70/10)	RAF1 (TKL/51/24)	MEK5 (STE/36/10)	AXL (TK/19/10)	FYN (TK/13/10)
**PDGFRB** (TK/69/11)	**RET** (TK/51/25)	**ABL1-nonphosphorylated** (TK/30/17)	ZAK (TKL/19/10)	**HCK** (TK/13/10)
**DDR1** (TK/64/16)	**FLT4** (TK/45/31)	**BRAF** (TKL/30/16)	BLK (TK/18/11)	**LYN** (TK/13/10)
**CSF1R** (TK/62/18)	**FLT3** (TKL/44/24)	**MERTK** (TK/28/20)	**EPHB6** (TK/18/11)	
**DDR2** (TK/ 59/21)	**VEGFR2** (TK/44/36)	**CIT** (AGC/26/15)	**SLK** (STE/18/11)	
**PDGFRA** (TK/55/25)	**LCK** (TK/41/21)	**TNNI3K** (TKL/23/18)	**STK36** (Other/16/13)	
**LOK** (STE/54/26)	**TIE1** (TK/37/31)	AURKC (Other/22/7)	YES (TK/16/13)	
**FLT1** (TK/53/27)	**FRK** (TK/36/26)	**CDK8** (CMGC/21/15)	ABL2 (TK/13/10)	

Kinases with a recorded positive biological affinity for Sorafenib are shown in bold.

Using data from LINCS, we analyzed a list of compounds with VEGFR2 activity based on KINOMEscan data. Among them, Sorafenib, the first compound in the list, was found to fit hypotheses **18** and **20**. Its KINOMEscan data, analyzed with the same cutoff as before, revealed activity on 104 kinases (442 kinases tested, with activity defined as percent ≤ 30%). Among the 35 kinases previously identified, 85% are also present in the 104 active kinases for Sorafenib (highlighted in bold in Table 9). Additionally, Sorafenib is listed in DrugBank^41^ as an inhibitor of KIT, FLT3, RET, VEGFR1, VEGFR2, VEGFR3, PDGFRB, and BRAF. Based on Kinobeads data, apparent k_d_^app^ values for Sorafenib are recorded for DDR1, DDR2, FLT3, MAP3K1, RET, and ZAK^34^.

Kinase data from ChEMBL were screened using hypotheses **16–20**, revealing that 17,468 compounds are able to fit at least one of them. This represents 13% of the overall dataset and covers 392 kinases with at least one associated compound. The hit percentage was half that observed with the eleven hypotheses. Yet we noted both stability and variations in compound/kinase associations, consistent with the hypothesis profiles.

Regarding the top five kinases previously identified with PKIS2 (see Table 9) and their corresponding results in ChEMBL, the percentage of fit for KIT is higher in ChEMBL compared to PKIS2 (see Table 10). The fit percentages for the other kinases also exceed the 13% average, with CSF1R showing a particularly high fit rate of 32%.

**Table 10:** Hit percentages for PKIS2 and ChEMBL (top five kinases in PKIS2).

	KIT	PDGFRB	DDR1	CSF1R	DDR2
PKIS2	32%	28%	38%	45%	45%
ChEMBL	40%	22%	18%	33%	28%

VEGFR2 remained logically the best kinase in terms of the number of associated compounds (Table 11). Serine/threonine-protein kinase B-RAF also maintained a high ranking with 34% of compounds fitting. We observed that the hit percentage for certain kinases decreased significantly compared to the analysis with the eleven DDR1 pharmacophores. For example, IGF1R dropped from 63% to 9%. In contrast other kinases like DYRK2, showed a notable increase, rising from 4% to 45% (548 compounds).

**Table 11:** Top 10 kinases matching the VEGFR2 pharmacophores ranked by the number of compounds.

Name	Family	Count	Min	Max	Percent
Vascular endothelial growth factor receptor 2	TYR	2642	7.39	10.68	29
Serine/threonine-protein kinase B-Raf	TKL	1401	7.62	10	34
MAP kinase ERK2	CMGC	1158	8.17	9.82	36
Nerve growth factor receptor Trk-A	TYR	959	7.02	9.4	26
MAP kinase p38 alpha	CMGC	850	7.09	10.4	19
Serine/threonine-protein kinase mTOR	PI3	814	7.59	10.1	18
Epidermal growth factor receptor erbB1	TYR	794	7.29	10.7	10
Tyrosine-protein kinase JAK2	TYR	776	8.26	10.35	12
Tyrosine-protein kinase receptor FLT3	TYR	757	7.61	10.82	23
Tyrosine-protein kinase JAK1	TYR	673	8.75	10.52	14

Count: Number of compounds; Family: kinase family; Min: Minimum activity; Max: Maximum activity ; Percent: percentage of compounds fitting the pharmacophore.

**Fig. 4:**
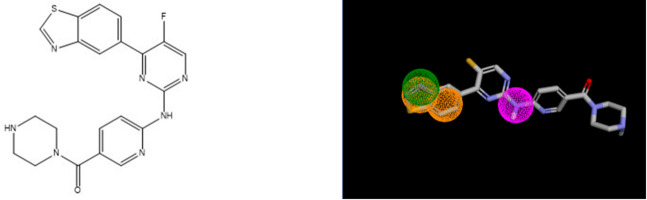
DYRK2 ligand with pharmacophore **16**.

An analysis on ChEMBL data specifically focused on DYRK2 (CHEMBL4376, IC_50_, 780 compounds) revealed that 289 compounds (37%) fit at least one of the five pharmacophores. Notably a large majority (281 compounds,36%) fit pharmacophore **16** (see Table 11). As an illustration, the most potent ligand, on DYRK2 (IC_50_ = 0,6 nM) fits perfectly the pharmacophore **16** (see Fig. 4).

**Fig. 5:**
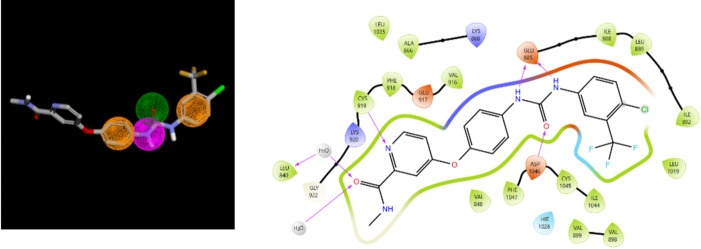
Hypothesis 20 fitted with Sorafenib (left). Ligand-protein interactions for the 3WZE complex generated using the Protein Preparation Workflow and the ligand interaction diagram in Maestro (Schrodinger Inc)).

From hypotheses **16-20**, we performed a screening on the ligands from the KLIFS database. Among the 4,731 ligand/kinase complexes in KLIFS, 27 correspond to VEGFR2_human. Our five hypotheses (**16**–**20**) fit 10 out of these 27 complexes (37%) and hypothese **20** fits the ligands of all ten complexes, including a complex with Sorafenib (3WZE^42^). In Fig. 5, the agreement between the hypothesis **20** and a type II kinase inhibition mode (DGF out and E from αC-helix) observed for Sorafenib is shown.

### Towards a kinase miner using pharmacophore hypotheses: DDR1 pharmacophores excluding VEGFR2

We tested a new screening approach by applying a set of criteria based on NEM values associated with different kinases for hypothesis selection. We extracted DDR1 hypotheses (NEM values ≥ 50 for DDR1) without VEGFR2 properties (NEM values ≤ 10 for VEGFR2). This approach resulted in 186 hypotheses, which were further refined into three MMRFS hypotheses **1**, **11**, **21** (see Table 4).

These three hypotheses **1**, **11**, **21** are associated with six kinases, representing 3.3% of the 178 kinases that have at least one hypothesis with NEM ≥ 50. Only hypothesis **21** is new compared to the previous eleven DDR1 hypotheses. On the six kinases, five belong to the TK family. DDR1 ranks first in the classification (DDR1 (TK/52/26)), covering 31% of active compounds and 5% of inactive compounds. Additionally, the top five kinases remain exactly the same as in the two previous cases (PDGFRB (TK/27/4 ; KIT (TK/25/6) ; CSF1R (TK/21/10) ; DDR2 (TK/21/10) ; RAF1 (TKL/21/10)).

From the three MMRFS hypotheses, a virtual screening on ChEMBL yielded a 9% hit rate, covering 362 kinases (12,231 compounds that were found to fit at least one of these hypotheses). When comparing the percentage of hits in function of the three different number of hypotheses, we observed a direct correlation (11/26%; 9/13%; 5/9%). While it is expected that the number of pharmacophores influences the number of hits, the strength of this correlation is surprising in this case.

Regarding the top five kinases identified in PKIS2 (see Table 12) and their corresponding results in ChEMBL, KIT, as observed previously, exhibits a higher hit percentage in ChEMBL than for PKIS2 (see Table 12).

**Table 12:** Hit percentages for PKIS2 and ChEMBL (top five kinases in PKIS2).

	KIT	PDGFRB	DDR1	CSF1R	DDR2
PKIS2	12%	11%	37%	17%	18%
ChEMBL	19%	5%	10%	5%	11%

**Fig. 6:**
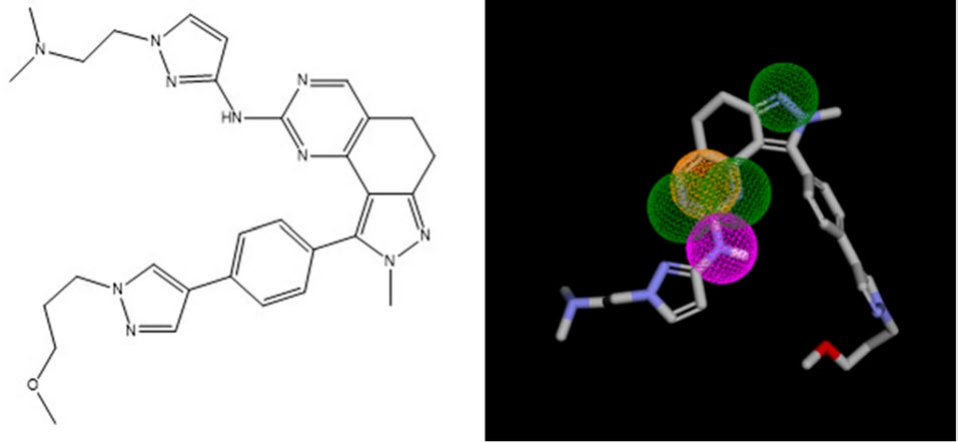
CHEMBL3979113 with pharmacophore **11**.

Insulin-like growth factor I receptor (IGF1R) emerges as to the top-ranked kinase (see Table 13) based on the number of compounds fitting these three hypotheses, placing it 9th in terms of hit percentage. This result is consistent with the findings from the first analysis using eleven pharmacophores because hypothesis **11** shows the strongest agreement with the 1244 compounds associated with IGF1R, covering 1153 compounds. A representative example is CHEMBL3979113 (IC_50_ = 0.25 nM**)**, which perfectly fits hypothesis **11** (see Fig. 6).

**Table 13:** Top 10 kinases matching the three hypotheses ranked by the number of compounds.

Name	Family	Count	Min	Max	Percent
Insulin-like growth factor I receptor	TYR	1244	7.75	10.3	49
Vascular endothelial growth factor receptor 2	TYR	931	6.80	9.34	10
Tyrosine-protein kinase BTK	TYR	792	7.54	10.85	23
Epidermal growth factor receptor erbB1	TYR	778	7.32	10.4	9
Tyrosine-protein kinase SYK	TYR	759	7.49	10.22	22
Serine/threonine-protein kinase B-raf	TKL	659	7.64	10.15	16
MAP kinase p38 alpha	CMGC	629	6.97	9.9	14
Tyrosine-protein kinase JAK2	TYR	404	7.12	10.4	6
Glycogen synthase kinase-3 beta	CMGC	401	6.91	9.55	10
Serine/threonine-protein kinase RAF	TKL	387	8.37	10.62	30

Count: Number of compounds; Family: kinase family; Min: Minimum activity; Max: Maximum activity ; Percent: percentage of compounds fitting the pharmacophore.

A screening was done with the hypothesis **21**, on the ligands from KLIFS (the two other led to no results). The compound described on Fig. 3 fits the hypothesis **21** and as is logically associated with 6HP9 complex for a type II kinase inhibition mode.

## Conclusion

We have developed a novel approach based on the definition of 3D pharmacophore hypotheses derived from a polypharmacological dataset to extract hypotheses associated with specific biological profiles. Starting from the core data of PKIS2, we successfully identified a set of pharmacophore hypotheses primarily related to the TK family, with a focus on DDR1 and VEGFR2 and particularly type II inhibitor interaction mode for the illustrations. Our findings are supported by biological data consistent with the predicted profiles.

We also explored the concept of a “kinase miner,” where hypotheses are associated with one kinase while being absent or minimally linked to another. While this approach is applicable to a broader subset of kinases, in this study, we concentrated on DDR1 and VEGFR2.

Beyond DDR1 and VEGFR2, the method can be applied to any kinase in PKIS2 or in other screening datasets. The software is open source and is designed to enable users to extend the analysis to their targets of interest. For prospective applications, any new ligand can be mapped onto the complete set of extracted hypotheses. Since each hypothesis has kinase-specific NEM annotations, matching a hypothesis directly provides a predicted activity profile for the new compound. By comparing which hypotheses are matched across a set of analogs, users can identify the pharmacophoric elements responsible for selective or polypharmacological behaviour. This makes the workflow applicable to any user-defined kinase profile.

The predictive potential of this initial molecular characterization based on pharmacophoric graphs will be more explored by incorporating GNN approach in the process.

Kinase gene symbols are reported using the approved nomenclature of the HUGO Gene Nomenclature Committee^43^ (see abbreviations).

## Data Availability

All datasets, source codes and results are available at https://doi.org/10.5281/zenodo.17698814.

## Abbreviations

AGC: Protein kinase A, G, and C family
CAMK: Calcium/calmodulin-dependent protein kinase family
ChEMBL: Chemistry European Molecular Biology Laboratory
CK1: Casein kinase 1 family
CMGC: CDK, MAPK, GSK, and CLK kinase family
DDR1: Discoidin domain-containing receptor 1
ECFP4: Extended-Connectivity FingerPrint (radius 4)
ETKDG: Experimental-torsion knowledge distance geometry
GR: Growth rate
HMS LINCS: Harvard Medical School Library of Integrated Network-Based Cellular Signatures
KLIFS: Kinase ligand interaction fingerprint and structure database
MMRFS: Maximal marginal relevance feature selection
NEM: Normalized enrichment measure
P_A_: Proportion of active compounds for a pharmacophore and a kinase
P_I_: Initial proportion of active compounds in PKIS2 for a kinase
PKIDB: Protein kinase inhibitor database
PKIS2: Published kinase inhibitor set 2
SMILES: Simplified molecular input line entry system
STE: Homologues of yeast sterile (STE) kinases
TK: Tyrosine kinase family
TKL: Tyrosine kinase-like family
VEGFR2: Vascular endothelial growth factor receptor 2 (VGFR2 also)
